# Methodology Used in Ecological Momentary Assessment Studies About Sedentary Behavior in Children, Adolescents, and Adults: Systematic Review Using the Checklist for Reporting Ecological Momentary Assessment Studies

**DOI:** 10.2196/11967

**Published:** 2019-05-15

**Authors:** Catiana Leila Possamai Romanzini, Marcelo Romanzini, Mariana Biagi Batista, Cynthia Correa Lopes Barbosa, Gabriela Blasquez Shigaki, Genevieve Dunton, Tyler Mason, Enio Ricardo Vaz Ronque

**Affiliations:** 1 Londrina State University Department of Physical Education Londrina Brazil; 2 Federal University of Mato Grosso do Sul Campus of Pantanal Corumbá Brazil; 3 Federal University of Technology of Paraná Academic Department of Humanities Apucarana Brazil; 4 Rio Preto University Center Department of Physical Education São José do Rio Preto Brazil; 5 Paulista University Department of Physical Education São José do Rio Preto Brazil; 6 University of Southern California Department of Preventive Medicine Los Angeles, CA United States

**Keywords:** physical activity, accelerometry, health behavior

## Abstract

**Background:**

The use of ecological momentary assessment (EMA) to measure sedentary behavior (SB) in children, adolescents, and adults can increase the understanding of the role of the context of SB in health outcomes.

**Objective:**

The aim of this study was to systematically review literature to describe EMA methodology used in studies on SB in youth and adults, verify how many studies adhere to the Methods aspect of the Checklist for Reporting EMA Studies (CREMAS), and detail measures used to assess SB and this associated context.

**Methods:**

A systematic literature review was conducted in the PubMed, Scopus, Web of Science, PsycINFO, Cumulative Index to Nursing and Allied Health Literature (CINAHL), and SPORTDiscus databases, covering the entire period of existence of the databases until January 2018.

**Results:**

This review presented information about the characteristics and methodology used in 21 articles that utilized EMA to measure SB in youth and adults. There were more studies conducted among youth compared with adults, and studies of youth included more waves and more participants (n=696) than studies with adults (n=97). Most studies (85.7%) adhered to the Methods aspect of the CREMAS. The main criteria used to measure SB in EMA were self-report (81%) with only 19% measuring SB using objective methods (eg, accelerometer). The main equipment to collect objective SB was the ActiGraph, and the cutoff point to define SB was <100 counts/min. Studies most commonly used a 15-min window to compare EMA and accelerometer data.

**Conclusions:**

The majority of studies in this review met minimum CREMAS criteria for studies conducted with EMA. Most studies measured SB with EMA self-report (n=17; 81.0%), and a few studies also used objective methods (n=4; 19%). The standardization of the 15-min window criteria to compare EMA and accelerometer data would lead to a comparison between these and new studies. New studies using EMA with mobile phones should be conducted as they can be considered an attractive method for capturing information about the specific context of SB activities of young people and adults in real time or very close to it.

## Introduction

### Background

Sedentary behavior (SB) is defined as any activity performed during awake time with low energy expenditure (below or equal to 1.5 metabolic equivalents) in a sitting or reclining position [1-3]. SB can be measured using subjective assessments (eg, questionnaires) [4] or using objective assessments (eg, accelerometers and inclinometers) [4]. Both methods of assessment have a number of strengths and weaknesses. Subjective measures of SB are able to distinguish between various types of SB (eg, watching television and internet use); however, subjective measures are prone to retrospective recall biases given that they depend on one’s ability to accurately recall previous SB [5]. Oppositely, objective measures of SB provide a fine-grained assessment of individuals’ level of SB but do not distinguish between different types of SB [5].

The discussion about the best method to measure SB is relevant because the more accurate information is collected about this behavior, the more precise information we can have about the context and the type of SB that the participant did, and thus better understand how this behavior can occur, distributed in different contexts throughout the day. Although the literature shows that the use of inclinometers [6] is the best method for measuring SB (because they can detect different postures adopted by subjects), more than half of studies (51%) that investigate physical activity (PA) and its domains still use ActiGraph monitors to obtain information about the total amount of time in a specific behavior [7].

Although both subjective and objective assessments are useful as measures of SB [8], they are not able to identify the context of the activities being performed by the subject [4]. Contextual factors include intrapersonal (eg, affect), interpersonal (eg, who one is with), and environmental factors (eg, location) in which SB occurs in daily life and are integral in understanding reasons for SB as well as outcomes of SB. Contextual factors of SB are often time-varying such that they change over the course of minutes, hours, or days. Owing to the time-varying nature of contextual factors, they cannot be adequately assessed using traditional survey measures. In addition, although survey measures of SB are able to capture information on types of SB, these measures are plagued by retrospective recall biases. Thus, despite researchers’ efforts to understand how SB is associated with health outcomes [9-12], there is still a gap in knowledge of the social and environmental context of SB in different populations and how context is associated with health outcomes [13].

The context of SB can be obtained through the use of questionnaires, but its application is burdensome for the participant and labor-intensive for the researcher and, in addition, may be inadequate for long-term monitoring studies [14]. Thus, it is highly likely that, in each of these contexts, there are distinct determinants for the subject to assume this behavior, as they are shaped by the attributes of the environments where they occur and the social structure involved [15].

Ecological momentary assessment (EMA) remedies many of the described limitations including identification of the type, environment, and context in which SB occurs as well as reduced reliance on retrospective recall [16]. EMA is an approach to collecting data in real time in individuals’ natural environment. Participants in EMA studies are instructed to respond to self-report surveys over the course of the day for a short period of time (eg, weeks and months) using an electronic device such as a mobile phone. Several methods may be used to collect EMA surveys including randomly signaling participants to complete a survey (ie, random interval contingent), having participants complete surveys at predefined intervals (ie, fixed interval contingent), or having participants complete surveys when a specific event occurs (ie, event contingent) [16].

To our knowledge, publications about PA using EMA have already been published among children and adolescents [17,18] and with adults [19]. However, considering that the importance of reducing time in SB has recently received significant attention and that the EMA is a relatively new methodology for investigating contexts of SB among children, adolescents, and adults, it is important to increase the understanding of best practices for using EMA to assess SB, mainly in the verification of association between context of SB and many health outcomes.

A primary advantage of using EMA to study SB is increased understanding of the context in which activities are being performed [5]. Thus, in addition to collecting the information that the subject was involved in a certain number of minutes at a given intensity of PA during the week, it is also possible to gain information about where and what type of activity this was, from the information recorded by the subject through EMA, which is very close to the moment when it happened [16,19]. More recently, research suggested the standardization of EMA use and proposed to study the Checklist for Reporting EMA Studies (CREMAS) for enhancing reliability, efficacy, and overall interpretation of the findings for future studies that use EMAs [20].

### Objectives

Therefore, the aim of this study was to systematically review the literature on EMA in SB by researching in children, adolescents, and adults, to verify the number of studies that adhere to the Methods aspect of the CREMAS, and to provide recommendations for measuring SB in EMA.

## Methods

### Information Sources

A systematic review of the literature was conducted in the following databases: PubMed, Scopus, Web of Science, PsycINFO, CINAHL, and SPORTDiscus, seeking to identify studies that used EMA to measure SB in children, adolescents, and adults. We considered an adolescent to be a person aged 10 to 19 years, as proposed by the World Health Organization.

The search comprised the entire period of existence of the databases until January 2018, in the English language. The search strategy used the following structure of keywords and Boolean operators: (“ecological momentary assessment” OR “EMA”) AND (“sedentary behavior” OR “sedentary behaviour”).

### Selection Criteria

The eligibility criteria for studies were as follows: (1) involved participants aged >8 years to <60 years; (2) not being a review or systematic review study; (3) used an EMA-based data collection method; and (4) focused on the assessment of SB. After the exclusion of studies according to the eligibility criteria, the remaining studies were analyzed by abstract or full-text reading and were excluded if they did not assess the main outcome (SB) via EMA. The selection of studies was conducted independently by 2 researchers (CLPR and ERVR). In case of inconsistency in the selection of records, a third researcher (MR) was invited to review the selection.

### Extraction Criteria

In total, 2 researchers independently extracted information from records about study characteristics including sample size, mean age, outcomes, and measures. Data extraction was also conducted for specific methods used in EMA studies from the CREMAS [20] including the main technology used, prompt approach (such as prompting design), wave duration, monitoring period, and prompt frequency. In addition, compliance (ie, overall response rate to EMA prompts) was extracted from studies. Finally, the authors detailed the measures used to assess SB, the context, and if it is the current or past measure. The authors choose to keep the records of different studies even when the overlapping of participants was found.

## Results

### Characteristics of the Studies

**Figure 1:**
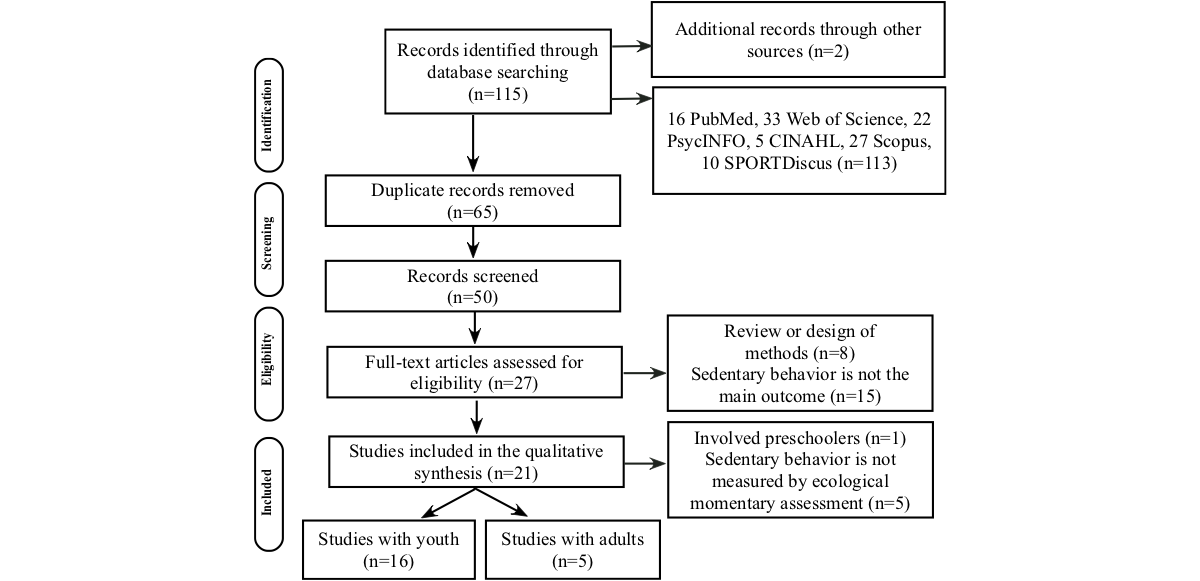
Preferred Reporting Items for Systematic Reviews and Meta-Analyses flow diagram.

A total of 115 potential studies were identified. Figure 1 presents the diagram of systematic reviews for analysis of studies proposed by the Preferred Reporting Items for Systematic Reviews and Meta-Analysis (PRISMA).

After the records were located in each of the databases, all were imported into the reference program. Following the PRISMA model, 65 duplicate records were excluded, after which the eligibility criteria were applied, and finally the analysis was carried out by reading the abstracts. Thus, 21 discrete studies were included in the qualitative synthesis of the data.

The characteristics for youth and adult studies are presented in Table 1. Of the 21 studies included in the qualitative synthesis, 16 were with youth [21,22,23-30,31-36] and 5 with adults [37-41].

The publication dates of articles ranged between 2007 and 2017 and 42.8% of these were published in the past 4 years. The majority of studies included both females and males; however, some collected information only with females [29,33,34] or only with males [30]. The mean number of participants per study was higher in studies with youth (696 participants) than in studies with adults (80 participants).

Only 3 studies collected information solely about SB [21,31,35], whereas the majority collected information about SB and other outcomes, such as PA, environmental factors, nutrition information, and depressive symptoms.

Only 1 study with youth [25] and 3 studies with adults [37,38,40] combined EMA with an objective measurement of SB (ie, accelerometers).

The methodological characteristics of the studies on SB in youth and adults are presented in Table 2.

**Table 1:** Characteristics of ecological momentary assessment studies on sedentary behavior in children, adolescents, and adults.

Study	N	Sample size		Age (years)		Outcome	Tools
		Girls	Boys	Mean (SD)	Range		
Gorely et al, 2007 [30]^a^	1371	850	521	14.7 (0.9)	13-16	PA^b^/SB^c^	EMA^d^ Diaries
Gorely et al, 2007 [29]^a^	923	923	0	14.7 (0.9)	13-16	PA/SB	EMA Diaries
Biddle et al, 2009 [21]^a^	1484	923	561	14.6 (0.9)	13-16	SB	EMA Diaries
Biddle et al, 2009 [22]^a^	991	606	385	14.1 (0.8)	13-16	PA/SB	EMA Diaries
Biddle et al, 2009 [23]^a^	1493	927	566	14.1 (0.8)	13-16	SB/other	EMA Diaries
Biddle et al, 2009 [24]^a^	623	376	247	15.5 (0.9)	13-18	PA/SB	EMA Diaries
Gorely et al, 2009 [27]^a^	1171	694	477	14.8 (0.8)	13-16	PA/SB	EMA Diaries
Gorely et al, 2009 [28]^a^	561	561	0	14.6 (0.8)	13-16	PA/SB	EMA Diaries
Dunton et al, 2011 [26]^a^	121	59	62	40.4 (9.7)	9-13	PA/SB	MP EMA^e^ and ACC (no measure SB)
Soos et al, 2012 [35]^a^	635	384	251	16.0 (1.0)	13-18	SB	EMA Diaries
Liao et al, 2014 [31]^a^	120	58	62	—^f^	9-13	SB	MP EMA
Soos et al, 2014 [36]^a^	812	464	348	15.6 (1.0)	9-13	PA/SB/other	EMA Diaries
Dunton et al, 2015 [25]^a^	200	200	0	—^f^	8-12	SB/other	MP EMA, questionnaire, and ACC
Raudsepp, 2016 [33]^a^	341	341	0	15.3 (3.0)	—^f^	SB/other	EMA Diaries and questionnaire
O’Connor et al, 2017 [32]^a^	175	91	84	9.6 (0.9)	8-12	SB/PA/other	MP EMA
Raudsepp and Riso, 2017 [34]^a^	122	122	0	11.4 (0.7)	11-12	SB/other	EMA Diaries and questionnaire
Rouse and Biddle, 2010 [41]^g^	84	38	46	19.5 (1.1)	—^f^	PA/SB	EMA Diaries
Dunton et al, 2012 [38]^g^	110	80	30	40.4 (9.7)	27-73	PA/SB	MP EMA and ACC^h^
Graves et al, 2015 [39]^g^	47	37	10	38.6 (9.5)	—^f^	SB/other	EMA Diaries, exams and questionnaires
Liao et al, 2015 [40]^g^	110	80	30	—^f^	27-73	PA/SB/other	MP EMA and ACC
Bruening et al, 2016 [37]^g^	133	97	36	18.8 (0.6)	—^f^	PA/SB/other	Automated Self-Administered 24-hour, Mobile ecological assessment, and ACC

^a^Studies with children and adolescents.

^b^PA: physical activity.

^c^SB: sedentary behavior.

^d^EMA: ecological momentary assessment.

^e^MP EMA: mobile phone ecological momentary assessment.

^f^Missing data.

^g^Studies with adults.

^h^ACC: accelerometer.

**Table 2:** Methodological characteristics of the ecological momentary assessment studies on sedentary behavior in children, adolescents, and adults.

Study	Technology; Response rates	Prompt design, Waves	Monitoring period, frequency	SB^a^ measures; Context of SB; Current, past SB
Gorely et al, 2007 [30]^b^	Paper and pencil diaries; 50.2%	RIC^c^, 2 times	4 days (3 vs 1), every 15 min	SR^d^ times per 15 min; 23 contexts; Current^e^
Gorely et al, 2007 [29]^b^	Paper and pencil diaries; 92%	RIC, 2 times	4 days (3 vs 1), every 15 min	SR times per 15 min; 23 contexts; Current^f^
Biddle et al, 2009 [21]^b^	Paper and pencil diaries; not informed	RIC, 2 times	4 days (3 vs 1), every 15 min	SR times per 15 min; 23 contexts by gender; Current^e^
Biddle et al, 2009 [22]^b^	Paper and pencil diaries; 95%	RIC, 2 times	4 days (3 vs 1), every 15 min	SR times per 15 min; 18 contexts; Current^f^
Biddle et al, 2009 [23]^b^	Paper and pencil diaries; 85%	RIC, 2 times	4 days (3 vs 1), every 15 min	SR times per 15 min; 22 contexts; Current^f^
Biddle et al, 2009 [24]^b^	Paper and pencil diaries; Hungary 96%, Romania 78%, Slovakia 86%	RIC	4 days (3 vs 1), every 15 min	SR times per 15 min; 23 contexts; Current^f^
Gorely et al, 2009 [27]^b^	Paper and pencil diaries; 50.2%	RIC, 2 times	4 days (3 vs 1), every 15 min	SR times per 15; 23 contexts by gender; Current^e^
Gorely et al, 2009 [28]^b^	Paper and pencil diaries; 91.4%	RIC, 2 times	4 days (3 vs 1), every 15 min	SR times per 15 min; 23 contexts; Current^f^
Dunton et al, 2011 [26]^b^	HTC Shadow MP^g^, MyExperience; 80% with ACC^h^	RIC, 1 time	4 days, (3 vs 7)	SR; 4 contexts; Current^f,i^
Soos et al, 2012 [35]^b^	Paper and pencil diaries; 75%	RIC	4 days (3 vs 1), every 15 min	SR times per 15 min; 22 contexts; Current^f^
Liao et al, 2014 [31]^b^	HTC Shadow MPMy Experience; 77%	RIC, 1 time	4 days, (3 vs 7)	SR; 2 contexts; Current^f^
Soos et al, 2014 [36]^b^	Paper and pencil diaries; 75%	RIC	4 days (3 vs 1), every 15 min	SR times per 15 min; 23 contexts; Current^f^
Dunton et al, 2015 [25]^b^	Mobile Phone Android or Motorola Moto G; Mothers 80%, Children 69%	RIC, 6 times	7 days (3 vs 7), 1 add for mothers	SR; 1 context; Current^f,j^
Raudsepp, 2016 [33]^b^	Paper and pencil diaries; 95.6%	RIC, 3 times	4 days (3 vs 1), every 15 min	SR times per 15 min; 4 contexts; Current^f^
O’Connor et al, 2017 [32]^b^	Mobile Phone Android or Motorola Moto G; not informed	RIC, 1 time	8 days (3 vs 4), 7 vs 8	SR; 1 context; Current^f^
Raudsepp and Riso, 2017 [34]^b^	Paper and pencil diaries; 81.8%	RIC, 4 times	4 days (3 vs 1), every 15 min add per day (feedback)	SR times per 15 min; 12 contexts; Current^f^
Rouse and Biddle, 2010 [41]^k^	Paper and pencil diaries; 57%	RIC, 1 time	2 days (1 vs 1), every 15 min	SR times per 15 min; 3 contexts; Current^e^
Dunton et al, 2012, 2015 [38]^k^	HTC Shadow MPMyExperience; 82%, 85% with ACC	RIC, 1 time	4 days, 8 per day	SR; 3 contexts; Current^f,l^
Graves et al, 2015 [39]^k^	Paper based EMA diaries; not informed	RIC, 1 time	5 days, every 15 min	SR times per 15 min; 1 context (sitting); Current^f^
Liao et al, 2015 [40]^k^	HTC Shadow MPMyExperience; 82%	RIC, 1 time	4 days, 8 per day	SR; 3 contexts; Current^f,m^
Bruening et al, 2016 [37]^k^	Android; iPhone Operational System, MP; Motorola Moto G; Mobile ecological momentary assessment, 84% with ACC	RIC, 1 time	4 days, 7 per day; 1 past per day	SR;10 contexts; Current^f,n^ and Past

^a^SB: sedentary behavior.

^b^Studies with children and adolescents.

^c^RIC: random interval contingent.

^d^SR: self-report.

^e^Location and company.

^f^Performing the behavior at this exact moment or very close to it.

^g^MP: mobile phone.

^h^ACC: accelerometer.

^i^Objectively ActiGraph GT2M (no measure SB)

^j^Objectively ActiGraph GT3X+<100 counts per min

^k^Studies with adults.

^l^Objectively; ActiGraph GTM2<100 counts per min/15 min interval

^m^Objectively; ActiGraph GTM2<100 counts per min/15-min window

^n^Objectively/ActiGraph GT3X+<100 counts per min/5 min prior EMA prompt

In relation to the Methods aspect of the CREMAS, 85.7% of the studies adhered to these items (18 studies of 21 met all the criteria; 3 studies did not mention wave duration). The most widely used software for mobile phone studies was My Experience [26,31,38,40]. One study used a version of an application (devilSPARC) created specifically for the study [37] and another 2 studies did not specify the software used [25,32]. The majority of studies included in this review (85.7%) presented information about response rates to EMA prompts, adhering to reporting recommendations. The mean range was between 50.2% and 95.6%.

All of the studies analyzed used the random interval contingent (EMA prompts were set to be randomized throughout the day) [20] to deliver the prompts. In general, studies with youth used multiple measurement waves of monitoring periods (ranging from 1 to 4 waves). All of the studies with adults used only one data collection wave.

Each wave ranged from 2 to 8 days of EMA monitoring. The most common prompting frequency was every 15 min mainly in studies with youth (75%). In studies with adults, 40% prompted at a frequency ≤ 15 min; another 40% prompted at a frequency ≥ 2 hours.

All articles used self-report measures to define different contexts of SB. The contexts utilized ranged from 1 to 23 different contexts. About 81% of the articles included questions related to the behavior of the moment, such as: *What are you doing now?*, *What are you doing and where are you?*, *What were you doing right before you got this text?*, *Which of these things have you done?*, *Who (if anyone) was with you while you were doing this?*, *What were you DOING right before the beep went off?*, and *Have you engaged in any of the following activities during the past two hours?*; 19% sought to identify the location and the company.

Only 4 studies (19%) used an objective method to measure SB, 1 with youth [25] and 3 with adults [37,38,40]. Although 1 study cited the use of an accelerometer, the main outcome (SB) was not measured with this method; only the total number of steps and moderate-to-vigorous PA were measured. In all cases, the accelerometer used was the ActiGraph (models GTM2 or GT3X+), and the cutoff point to define SB was <100 counts/min. A 15-min window for more and less of the registration prompt obtained by EMA was the most common.

## Discussion

The aim of this study was to systematically review the literature on EMA studies of SB in youth and adults, verify the number of studies that adhered to the Methods aspect of the CREMAS, and describe the measures used to assess SB in EMA. There was a paucity of EMA studies of SB in adults (n=5), indicating a need for more research in this area. The majority of studies were conducted with youth, and these studies typically collected more waves of data and had larger sample sizes compared with studies in adults. In general, the samples in the reviewed studies often had a greater number of women compared with men.

### Principal Findings

Related to the studies conducted with mobile phones, the most commonly used software was MyExperience. This was one of the first software programs developed for EMA and it is an open license software developed especially for Windows mobile devices [42]. However, MyExperience was used in older studies and currently newer technology is available for use in EMA studies. One factor to consider when choosing software is accessibility on various mobile operating systems—some are only available for use on Android and others use Android and iPhone Operational System. Another consideration is cost of the software, which may limit applicability for large-scale studies [4].

This review indicated that EMA monitoring periods lasted from 4 to 8 days, 4 days being the most cited for capturing behavioral information.

Although the use concomitant of EMA in mobile phones and accelerometers can represent the best method to measure SB, we believe that 4 days of concomitant use of EMA and an accelerometer is a good recommendation to obtain more accurate information on the context and pattern of SB, representing a typical week of this behavior.

### Comparison With Previous Work

The fact that the subject is monitored several times during the day may induce them not to respond to the activity being performed at the time of prompting; however, this is a limitation not only of this type of record, but also of self-reporting instruments and questionnaires [4]. On the contrary, the use of EMA could also lead to decreased burden on the participant compared with the use of questionnaires, potentially yielding higher rates of compliance and lower rates of missing data [37]. In general, the response rates in studies that use EMA to assess with mobile phones are very high [25,37,38,40], providing evidence of the feasibility of using EMA to collect information about SB. This could be because of the easy access to mobile phones nowadays.

In our systematic review, we only identified 3 studies with adults that combined EMA with mobile phones and accelerometers [37,38,40]. All of these indicated the acceptability of 4 days of use of the EMA protocol in mobile phones to measure SB in adults and that this method can offer an innovative approach to capture PA and the context of SB. For purposes of comparison of the records between EMA and the accelerometer, it might be useful to standardize the 2 pieces of information to the same number of days.

This analysis may provide researchers with a more accurate interpretation of the context in which each sedentary period occurs, as well as identification of the intensity of PA that the subject assumes when he or she interrupts SB [43], for example. Moreover, it could help to eliminate the information bias of subjective instruments, which depend on the subject’s capacity to remember what they did at a particular moment of the day, as it has already been found that there is great variation between subjective and objective measures [44].

Identification of the context of the activities is important, as, in addition to the term *sedentary behavior*, there are particular SBs that generally occur in a variety of contexts such as watching television and other recreational activities with a screen in the home environment and occupations that require prolonged sitting in work and transport environments [45]. Thus, it is highly likely that in each of these contexts, there are distinct determinants for the subject to assume this behavior, as they are shaped by the attributes of the environments where they occur and the social structure involved [45]. In addition, a recent study showed that time watching television has a stronger magnitude of effect on all-cause mortality [15]. In this sense, the use of EMA can provide this identification about the context, and combined with information of the accelerometer, we can estimate the time in which the subject can spend in a specific type of SB context. This kind of information has not yet been researched and gains a body of evidence to future research.

Recently, more advantages have been highlighted with regard to the use of mobile phones, such as collecting data more quickly from a large number of people than traditional cross-sectional or other methods. In addition, this paper introduces new concepts that must be explored in the field of research on PA behavior, which relate to synchrony, sequentiality, and instability [46].

In total, 2 systematic reviews, aimed at providing an overview of existing studies on sedentary time in children and adolescents [47] and in adults [48], through a joint programming initiative called DEterminants of DIet and Physical ACtivity [49], made important notes on the need for harmonization and standardization of methods to assess sedentary time in this population, mainly in combination with objective and self-report methods.

In this sense and seeing that the evidence indicates that the use of EMA is still limited in the adult population, further studies should be conducted, as, in this population, life contexts can lead to an even greater amount of SB involvement owing to commitments such as transportation and work. Thus, as SB can be identified in different contexts, new studies can also be conducted that identify which of these may eventually have the greatest impact on health risks. In addition, new research could advance the comparative analysis of SB measurements obtained by EMA and objective measures such as accelerometry, or analyze the posture adopted by participants (inclinometers).

### Limitations

This review has strengths and limitations. The strength of this review is that it is the first to systematically review the literature focusing on EMA studies of SB. In addition, this is the first systematic review that used the CREMAS specifically to identify methodological characteristics of the EMA studies [20], demonstrating the importance of tailoring methods to the unique futures of EMA studies, especially those using mobile phone technology. Although an exhaustive literature search was conducted, it is possible that some studies may have been missed and some details about quantitative study characteristics may have been omitted.

### Conclusions

This review systematically evaluated information about the characteristics and methodology of empirical articles using EMA to measure SB in youth and adults. The majority of studies adequately presented the minimum criteria necessary for describing studies conducted with EMA, as proposed in the CREMAS. The main assessment used to measure SB with EMA was self-report; only a few studies used objective methods (accelerometer). There was no single standard adopted in the included studies to compare the EMA and accelerometer data, but we believe that the standardization of criteria (eg, use of a 15-min window to compare EMA and accelerometer and the use of the same cutoff point to define SB such as <100 counts/min) lead to higher quality studies. New studies using mobile phones should be carried out as they can be considered an attractive method for capturing information about the specific context of SB activities of youth and adults, in real time or very close to it.

## Abbreviations

CREMAS: Checklist for Reporting EMA Studies
EMA: ecological momentary assessment
PA: physical activity
PRISMA: Preferred Reporting Items for Systematic Reviews and Meta-Analyses
SB: sedentary behavior
